# The Dual Role of Echocardiography in the Diagnosis of Acute Cardiac Complications and Treatment Monitoring for Coronavirus Disease 2019 (COVID-19)

**DOI:** 10.3389/fcvm.2020.00129

**Published:** 2020-09-02

**Authors:** Agathi-Rosa Vrettou, John Parissis, Ignatios Ikonomidis

**Affiliations:** 2nd Department of Cardiology, COVID-19 Infection Reference Center, Attikon University Hospital, National and Kapodistrian University of Athens, Athens, Greece

**Keywords:** coronavirus, transthoracic echo, Coronavirus disease 2019, SARS-CoV 2, global longitudinal strain, antiviral treatment, anti-inflammatory treatment

## Abstract

The Coronavirus Disease 2019 (COVID-19) pandemic, being caused by an easily and rapidly spreading novel betacoronavirus, has created a state of emergency for people, the scientific community, healthcare systems and states, while the global financial consequences are still unfolding. Cardiovascular complications have been reported for COVID-19-infected patients and are associated with a worse prognosis. ECG and biomarkers may raise suspicion of cardiac involvement. However, transthoracic echocardiography is a fast and reliable bedside method to establish the diagnosis of cardiac complications, including acute coronary syndromes, pericarditis, myocarditis, and pulmonary embolism. Early detection of cardiac dysfunction by speckle tracking echocardiography during off-line analysis may be used to identify a high-risk population for development of heart failure in the acute setting. Precautionary measures are mandatory for operators and equipment to avoid viral dispersion. No specific treatment is yet available for severe acute respiratory syndrome coronavirus 2 (SARS-CoV 2), and a variety of antiviral, immune-modifying, and antioxidant agents are therefore under intense investigation. Echocardiography, including assessment of myocardial deformation, may provide a useful tool to monitor the effects of the various treatment regimens on cardiac function both acutely and in the midterm.

## Introduction

A novel enveloped, single-stranded, positive-sense RNA betacoronavirus belonging to the family of coronaviruses has been identified as the causative agent of the novel viral pneumonia that started in the city of Wuhan, Hubei Province, China, on December 12, 2019, (1) and has turned into a global health emergency. The Coronavirus Disease 2019 pandemic counts, as of June 4, 2020, over 6.5 million confirmed cases in 188 countries and regions in the world and 384.815 fatalities (2), rapidly doubling the number of deaths within a month. The consequences of the pandemic in terms of its effects on the world population and global economy are still unfolding. The disease varies considerably from an asymptomatic or mild form without pneumonia to mild forms of pneumonia to severe pneumonia with lung consolidation that can lead to respiratory failure, sepsis, and multiorgan failure (3). Compared to the previous two coronaviruses that cause severe disease in humans, SARS (Severe Acute Respiratory Syndrome) and MERS (Middle East Respiratory Syndrome), SARS exhibits environmental stability (4) and MERS requires close and prolong contact for contamination (5), while the current coronavirus shows easy and high transmissibility, partly related to the high viral load early in the course of the disease (6).

Cardiovascular complications are relatively common, occurring in up to 25% of COVID-19 patients (7, 8) (Table 1). Myocardial injury is associated with a 37% in-hospital mortality even in patients without prior cardiovascular disease (9, 15). Cases of acute myocarditis have been reported presenting either as fulminant myocarditis or with symptoms mimicking an acute coronary syndrome (ACS) (16, 17). Pathology evidence of myocardial infiltration by a limited number of monocytes, lymphocytes, and/or neutrophils (18), and rarely associated epicarditis (19) may be suggestive of either activation of the systemic immune response or myocardial inflammatory infiltration due to viral-induced myocyte lysis. Patients presenting with ST elevation myocardial infarction (STEMI) either as an initial manifestation of the disease or during the course of hospitalization for COVID-19 disease (20) were treated with primary percutaneous intervention (PCI). Interestingly, 39% of patients had no evidence of obstructive coronary artery disease on coronary angiography, a finding that questions thrombolysis as a therapeutic alternative to timely coronary angiography and possibly primary PCI. Myocardial injury (11, 14) may also be attributed to myocardial supply/demand mismatch precipitated by hypoxemia, hypotension, tachycardia, and an uncontrolled inflammatory response, leading to cytokine release syndrome (10).

**Table 1:** Cardiovascular complications in COVID 19 hospitalized patients in selected studies.

**Study**	**City, Country**	**Total study population**	**Acute myocarditis**	**DVT /Pulmonary embolism**	Acute cardiac injury^*^	**ACS**	**Ischemic stroke**
Huang et al. (6)	Wuhan, China	41 patients	–	–	5/41 (12%)		
Yang et al. (7)	Wuhan, China	52 critically ill ICU patients	–	–	12/52 (23%)		
Shi et al. (8)	Wuhan, China	416 hospitalized patients	–	–	82/416 (19.7%)		
Guao et al. (9)	Wuhan, China	187 hospitalized patients	-	-	52/187 (27.8%)		
Middeldorp et al. (10)	Amsterdam, the Netherlands	198 hospitalized patients	–	39/198 (20%)13% DVT 6.6%PE			
Bombard et al. (11)	Paris, France	135 patients	–	32/135 (24% PE)			
Lodigiani et al. (12)	Milan, Italy	388 hospitalized patients	–	26/388 (6.7%) 16/388 (4.4% VTE)10/388 (2.8% PE)		4/388 (1.1%)	9/388 (2.5%)
Inciardi et al. (13)	Brescia Lombardy Italy	99 hospitalized patients	–	12/99 (12%)		3/99 Arterial thromboembolism	
Chen et al. (14)	Wuhan, China	274 hospitalized patients	–		89/203 (44%)		

* *hs Trop≥99^th^ percentile, ECG changes, Echocardiography abnormalities*.

Pulmonary embolism (12, 13, 21, 22) occurs frequently occurring in up to a quarter of all COVID-19 patients despite prophylactic antithrombotic treatment. The activation of the coagulation cascade by inflammatory cytokines, direct endothelial injury of lung microcirculation, antiplatelet activation, and suppression of the fibrinolytic system are all involved synergistically in the mechanism of venous thrombosis (23). Sudden hemodynamic compromise, the need for increased oxygen supplementation in discordance with radiological disease severity or elevations in D-dimers, especially >1 g/l, should prompt further diagnostic work-up with Computed Tomography Pulmonary Angiography (CTPA) to confirm pulmonary embolism.

## Echocardiography for the Diagnosis of Acute Cardiovascular Complications During the Course of COVID-19

**Figure 1:**
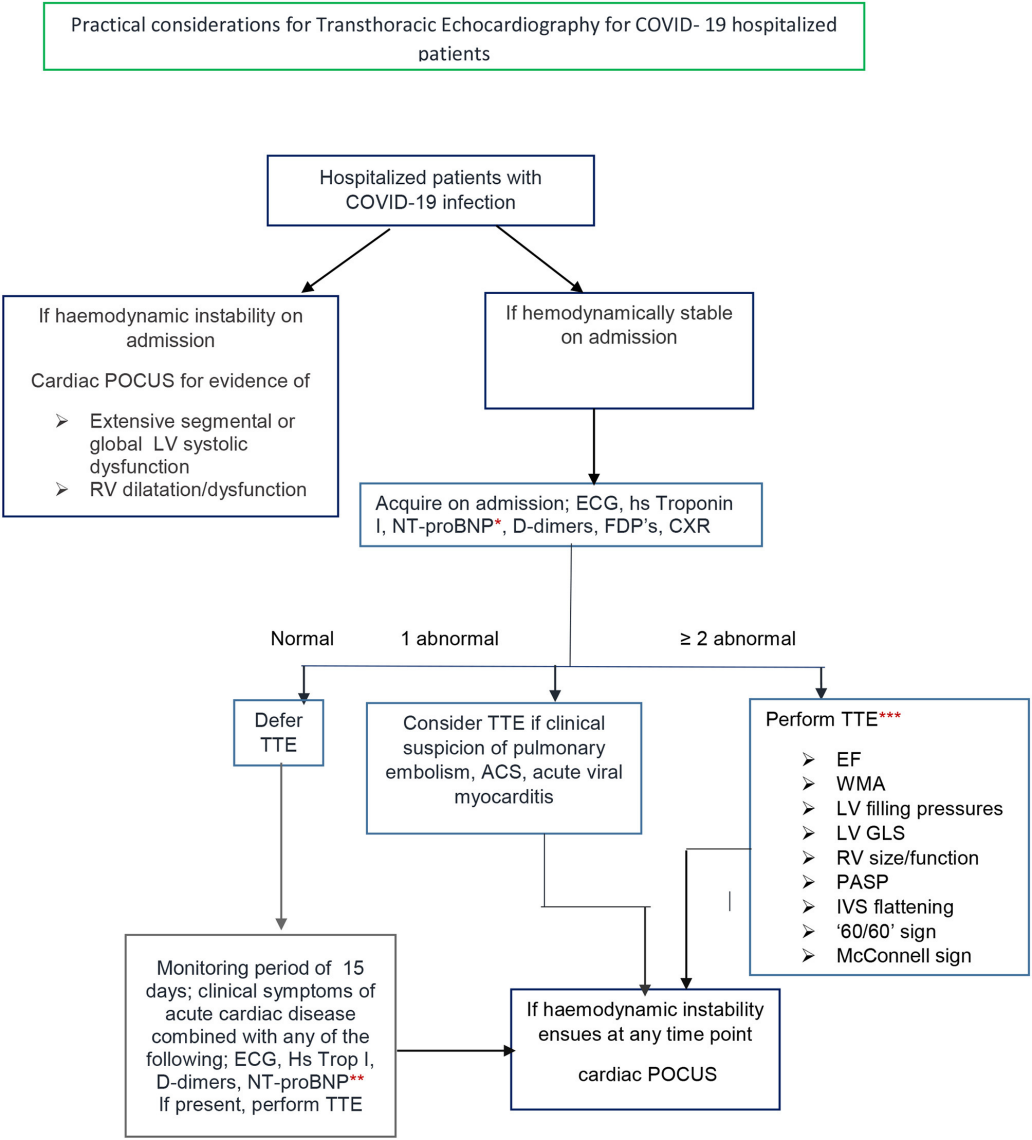
Transthoracic echocardiography for hospitalized COVID 19 disease patients. ^*^NT-pro BNP level on admission ≥300 pg/ml (9). ^**^NT-pro BNP level during hospitalization ≥940 pg/ml (16, 17). ^***^One experienced sonographer/cardiologist. Use PPE. Disinfect equipment in and out of the ward. EF, ejection fraction; WMA, wall motion abnormality; LV, left ventricle; RV, right ventricle, IVS, interventricular septum; GLS, global longitudinal strain by offline analysis; Cardiac POCUS, cardiac point of care cardiac ultrasound.

Echocardiography is a first-line imaging method to diagnose overt and subtle myocardial dysfunction (14). Localized wall motion abnormalities may be suggestive of a culprit coronary artery lesion leading to an ACS, whereas a diffuse pattern of abnormal segmental longitudinal myocardial strain by echocardiography may support the diagnosis of myocarditis over this of an acute coronary syndrome. Signs indicative of acute pulmonary embolism (PE) should be sought. Right ventricular dilatation from a PLAX view or a basal RV/LV ratio > 1 in a four chamber view can be easily measured as well as pulmonary artery diameter from a short axis view. RV dysfunction can be assessed both qualitatively and with the integration of simple and fast measurements of TDI and TAPSE. Right ventricular systolic hypokinesis is associated with worse 30–day prognosis (24). The presence of a Mc-ConnelI sign increases the sensitivity for the diagnosis though specificity on its own is only 33% (25). Echocardiographic evidence of RV pressure overload expressed by systolic and diastolic septal flattening can be noticed (26). Estimation of pulmonary artery systolic pressure from tricuspid systolic gradient and a usually increased inferior vena cava diameter combined with a short acceleration time and midsystolic notch in the PW Doppler of the RVOT(‘60/60; sign) (26) further indicates increased PA pressure and proximal thromboemboli (27). In clinical practice, hemodynamic instability should lead to a cardiac Point of care Ultrasound (POCUS) (28, 29) to determine the presence of left ventricular dysfunction and/or right ventricular dilatation or a large pericardial effusion (Figure 1). Handheld echo devices are the most suitable for this indication as they are portable, more easily disinfected compared to traditional ultrasound machines, while images can be stored and transferred to a PC. On the other hand, in an hemodynamically stable patient with COVID-19 infection, a high clinical suspicion of cardiac involvement, supported by one abnormal diagnostic parameter acquired on admission-that is ECG, CXR, hs Troponin I, NT-proBNP, D-dimers, FDP's—should lead to consideration of a transthoracic echocardiogram (TTE). Solely one abnormal laboratory test cannot be the only criterion for a TTE in a stable COVID-19 infection patient, since their positive predictive value for a specific disease may be low, especially in patients with concomitant chronic diseases and thus lead to a TTE patients' without cardiac involvement. For example, D-dimers may be elevated in various diseases where activation of coagulation and fibrinolysis is present such as cancer patients with COVID 19 (30) or chronic kidney disease (31). For NT-pro BNP values, a level greater that 300 pg/ml (32) should be considered as abnormal. Although, when taking age into consideration, a higher NTproBNP level >1,800 pg/ml should be used to suspect acute heart failure in patients older than >75 years, (33, 34), anything lower than those NT pro-BNP values, at a cut-off level of 940 pg/ml, has been related to adverse outcomes in critically ill patients admitted to ICU (35). Elevated hs cardiac Troponin I is even more specific to myocardial injury than CTnT (36) and may be attributed to multiple and overlapping mechanisms. Cardiac Troponin I can be measured on admission and during hospitalization of COVID- 19 patients in conjunction to NT-pro BNP and thus guide the need for TTE.

On the contrary, when two of the initial diagnostic parameters are abnormal, a complete TTE should be performed to diagnose possible cardiac involvement and ventricular dysfunction. Even the combination of two elevated laboratory biomarkers alone, should lead to a complete TTE, as hsTrop T and NT-proBNP levels were linearly correlated and considerably increased in non-survivor COVID-19 patients (15). As cardiac complications may occur within 15 days of admission monitoring of NT-proBNP, troponins and D-dimers in combination with the patients' clinical status are recommended throughout this period (37).

Notable considerations exist when estimating left and right ventricular systolic function, one being the presence of tachycardia, related to numerous factors such as fever, hypoxemia, cytokine production, and systemic inflammation. Ventricular systolic function is negatively affected in the presence of tachycardia due to the force–frequency relationship. Diastolic function estimations parameters are also affected by tachycardia, since fusion of transmitral E and A waves makes estimation of their ratio and the DT time inaccurate (38). In that case, a TR maximum velocity jet for estimation of PA systolic pressure may be an indicator of LV filling pressures. An impaired global longitudinal strain of the LV or RV may also indicate the initiation of myocardial damage particularly in patients with elevated troponins.

Echocardiography exams should be performed by experienced practitioners to ensure quick acquisition of high quality images (24) and thus minimize possible viral exposure.

TEE carries a high risk of spreading aerosolized viral material within an exam environment. It should be avoided during the pandemic (39). It should be only carried out when there is an absolute indication (e.g., bacterial endocarditis), and the results are expected to modify patient's management. In that case, the exam should be carefully designed by the patient's medical team (40).

## Considerations for Health Care Professionals

Safety is of utmost importance for the personnel involved in echocardiography of suspected or confirmed COVID-19 patients. Frequent and meticulous handwashing is mandatory. Personal Protective Equipment (PPE) should be used depending on the risk level. Face masks, headcovers, eye shields, gloves, gowns, and shoe covers should be used when examining high risk patients. Detailed description for the PPE is provided by WHO, ASE and EACVI (24, 34, 41). Institutions provide their own detailed protocols in line with international societies' guidance and local experience.

## Considerations for Equipment Disinfection

Equipment used for Echocardiography studies should be thoroughly disinfected at the end of the exam, in the examination room and again at the hallway (8). Dedicated machines for scanning suspected or confirmed patients may be preferable at this time. Manufacturer's guidance for proper disinfection of the different types of machines should be followed as well as instructions given by certain disinfectant producers.

## Echocardiography for Monitoring Treatment Effect on Myocardial Function

A number of different pharmacological agents (42)—antivirals, investigational antivirals, and immune-system-mediating agents—are currently under investigation for COVID-19 treatment in 1,833 clinical trials enrolled at Clinical Trials.gov as of May 30, 2020, under the search terms COVID-19 and SARS-CoV-2.

Chloroquine and hydroxychloroquine are used as antimalarial chemotherapeutic agents. They are also used in the treatment of different autoimmune diseases due to their multitargeted mechanism of action. They inhibit release of inflammatory cytokines by mononuclear cells (43) and interfere with Toll-like receptor signaling pathways and cyclic GMP-AMP (cGamP) synthase (cGaS) activity. In COVID-19 infection, it has been shown *in vitro* that chloroquine can inhibit viral binding to ACE2 receptor (44). They are both contraindicated in G6PD deficiency. QT prolongation and possible TdP may occur, especially in patients with hypokalemia, hypomagnesemia, hypocalcemia, or on concomitant use of QT prolonging drugs. Serious cardiac side effects occur especially in high cumulative doses after long term treatment, though low cumulative doses (45) may also result in heart failure. Conduction disorders were the main side effect reported in a systematic review (38), affecting 85% of patients. Other non-specific adverse cardiac events include ventricular hypertrophy (22%), hypokinesia (9.4%), heart failure (26.8%), pulmonary arterial hypertension (3.9%), and valvular dysfunction (7.1%), which can be readily ruled in by echocardiography (37). Both agents increase the bioavailability of metoprolol via inhibition of CYP2D6-catalyzed pathways (46). Frequent ECG is recommended, while TTE may reveal early myocardial dysfunction leading to possible treatment discontinuation. A number of ongoing clinical trials (47, 48) examine the therapeutic benefit hydroxychloroquine in COVID-19-infected patients as well as its role in chemoprophylaxis for exposed healthcare workers (49).

Recombinant human angiotensin converting enzyme 2 (ACE−2) has experimental (50) and clinical data (51) on the attenuation of acute lung injury by lessening angiotensin II levels and possibly IL-6.

Convalescent plasma treatment may be promising in terms of viral load and even mortality (52).

Corticosteroids have conflicting evidence for their effect on SARS CO-V 2 infection as they may delay viral clearance from blood and respiratory tract based on data from previous coronaviruses outbreaks (53). On the contrary, a small retrospective clinical trial of early, low-dose, short-term administration of methylprednisolone was associated with improved outcomes in patients with COVID 19 pneumonia (54), revealing the need for further clinical studies.

Remdesevir, a nucleotide analog inhibiting viral RNA polymerases, has an Emergency Use Authorization (EUA) from FDA for suspected or confirmed COVID 19 adult and children patients with severe disease since May 2020 (55). It has been shown to inhibit SARS and MERS in an *in vitro* model of human epithelial airway cells (56) and is also under clinical investigation (57–60). Preliminary results in limited number of patients point to further clinical studies (61).

The combination lopinavir/ritonavir is used in treating HIV-1 infection—they are both aspartase protease inhibitors, and ritonavir increases its plasma half-life. This drug combination has been reported to reduce viral load in clinical case reports (62), although the first clinical trial did not show statistically significant benefit (63). HAART (Highly Active Antiretroviral Treatment), especially protease inhibitors, have been associated with endothelial dysfunction and subclinical atherosclerosis (64–66). HAART may promote metabolic factors such as hyperlipidemia and induce atherosclerotic lesion formation through a CD-36 dependent accumulation of cholesterol in macrophages (30, 67, 68). These mechanisms, combined with the possible myocardial injury associated with the infection itself (9), may contribute to vascular and myocardial dysfunction. Therefore, for patients that have recovered from SARS-CoV-2 infection under protease inhibitor treatment, vascular, and ventricular function should be assessed by TTE at the end of the treatment and possibly at a 3- to 6-month intervals. The combination can also promote QT and PR interval prolongation as well as second and third degree AV block (11). Moreover, lopinavir/ritonavir are CYP3A4 inhibitors. They therefore cannot be used concomitantly with chloroquine (69), while antiplatelet and anticoagulant drugs may need dose adjustment or monitoring (59). Combination therapy of lopinavir /ritonavir, ribavirin and interferon b-1b was superior to lopinavir/ritonavir in a phase 2 clinical trial in terms of symptom alleviation, viral shedding, and hospital stay (70).

Monoclonal antibodies, such as tocilizumab (71), sarilumab (72), and bevasizumab (73), are under investigation to control the cytokine surge associated with the severe form of COVID-19 infection manifested as acute respiratory distress syndrome and multiorgan failure. IL-6 inhibition with biological agents such as tocilizumab and sarilumab may show a beneficial effect in controlling the excessive cytokine production (74) and evolution to alveoli consolidation. As has been recently shown in mechanically ventilated patients with COVID-19 infection (75), excessive IL-6 production is associated with lymphopenia and immunoparesis as assessed by low expression of the humanleukocyte antigen (HLA)-DR on CD14-monocytes, and this effect is reversed by tocilizumab. Additionally, IL-1b production is major factor contributing to the macrophage activation syndrome (Haemophagocytic lymphohistiocytosis syndrome) which characterizes significant number of the critically ill COVID-19 infected patients. Another possible mechanism for monoclonal antibodies beneficial effect could be mediated by preserving endothelial glycocalyx integrity. Damage of endothelial glycocalyx increases vascular permeability to circulating blood cell inflammatory markers and proteins (76) and may thus mediate lung injury and initiate SARS in COVID-19 as has been previously shown in septic patients. Anti-inflammatory treatment may exert beneficial effect on endothelial glycocalyx and thus may offer protection from evolution to alveoli exudation (77). Moreover, anti-inflammatory treatment with tocilizumab but also anakinra—an IL 1 receptor antagonist—exhibit beneficial effects on vascular function and myocardial function (78), as has been shown in patients with rheumatoid arthritis.

Additional protective mechanisms for IL-6 and IL-1 inhibitors may be related to regulation of ROS production, which hampers cellular functions, such as with the proteasome, leading to impaired endogenous protein degradation and mitochondrial dysfunction, augmenting the damage promoted by the direct interaction of SARS-COV proteins with the proteasome (79). ROS may activate the STAT/IL-6 axis (80) and promote IL-8 expression in pulmonary epithelial cells stimulated with lipid-associated membrane proteins from Mycoplasma pneumonia (81), triggering cytokine release and immune cell infiltration in the lung cells. Agents with inherent antioxidant properties such as N-acetylcysteine (NAC) and vitamin C may also be shown to be effective. The beneficial anti-inflammatory effect of monoclonal antibody treatment on myocardial function in COVID-19 infected patients may by easily monitored by an improvement in global longitudinal strain (GLS) toward normal values (81), as has been previously shown in patients with rheumatoid arthritis and uncontrolled inflammation (78, 79). The lowest expected normal values for GLS are −16.7% in men and −17.8% in women, according to a recently proposed consensus document, and these are similar to the values reported after remission of the acute inflammatory exacerbations by biological agents in patients with rheumatoid arthritis (78, 79).

## Conclusion

The COVID-19 pandemic, still unfolding around the world, has created a significant worldwide human, scientific, financial, and psychological burden, requiring innovative and cooperative strategies to combat the pandemic and its unprecedented consequences. TTE is required to guide clinical management of patients with abnormal ECG and/or biomarkers, as it may diagnose early cardiac involvement in the acute setting. Antiviral, anti-inflammatory, and antioxidant treatment agents as well as hyperimmune plasma are being investigated in a multitude of clinical trials. Echocardiography provides a valid method to monitor myocardial effect of potential treatments for COVID-19 during hospitalization and in the mid-term follow up.

## Data Availability Statement

The original contributions presented in the study are included in the article/supplementary material, further inquiries can be directed to the corresponding authors.

## Author Contributions

II had the original idea of the manuscript. JP reviewed the biomarkers and treatment section. A-RV wrote the initial version of the manuscript including figure. All authors offered comments on the manuscript's sections.

## Conflict of Interest

The authors declare that the research was conducted in the absence of any commercial or financial relationships that could be construed as a potential conflict of interest.
